# Recruitment and retention of clinical trial participants: understanding motivations of patients with chronic pain and other populations

**DOI:** 10.3389/fpain.2023.1330937

**Published:** 2024-03-28

**Authors:** Joyce K. Anastasi, Bernadette Capili, Margaret Norton, Donald J. McMahon, Karen Marder

**Affiliations:** ^1^ Division of Special Studies in Symptom Management, New York University, New York, NY, United States; ^2^ Heilbrunn Family Center for Research Nursing, The Rockefeller University, New York, NY, United States; ^3^ Department of Nursing, St. Joseph's University, Brooklyn, NY, United States; ^4^ Irving Medical Center, Columbia University, New York, NY, United States

**Keywords:** clinical trials, recruitment, retention, study participants, chronic pain, chronic conditions

## Abstract

This paper aims to present and discuss the issues, challenges, and strategies related to recruitment and retention in clinical trials involving participants with chronic pain. The randomized controlled clinical trial (RCT) is widely regarded as the gold standard for evaluating clinical interventions. However, it is crucial to acknowledge and address the challenges associated with recruiting and retaining participants. To prioritize the experience of the study population, targeted outreach strategies and a patient-centric approach are necessary. Researchers should consider incorporating recruitment and retention strategies during the study design phase. Implementing multi-pronged recruitment methods, leveraging relationships with community providers, and involving representatives of the patient population are helpful approaches. Effective communication and maintaining a professional environment are vital for optimizing engagement and supporting the successful execution of clinical trials involving participants with chronic pain.

## Introduction

The prospective clinical trial, specifically the randomized controlled clinical trial (RCT), is the gold standard for evaluating clinical interventions. Sufficient recruitment enables a trial to get underway, and retention allows it to proceed to completion, yield outcomes that may be reliably interpreted, and expand the knowledge base to benefit future patients. Recruitment and retention of participants are vital to clinical trial success as they enable the statistical power to detect an intervention's effect.

Recruitment and retention are notoriously challenging (1). Researchers rank recruitment of clinical trial participants as the most common challenge they face after receiving funding (2). A review of the United Kingdom showed that nearly one-third of publicly funded RCTs met with significant recruitment obstacles that required modification of initial goals and/or extension of the enrollment period (3). Drop-out rates of 25%–30% are common, and rates up to 70% have been reported (1, 4). Potential consequences of poor participant recruitment or retention include delayed timelines, increased costs, questionable results, and study discontinuation or failure (5, 6).

Therefore, clinical investigators must devote significant energy to recruitment and retention efforts in clinical trials, ideally starting before the design phase and continuing through participants' final follow-up visits. Investigators should understand factors influencing patients' decision to enroll in clinical trials and the ability and willingness to stay engaged. They should be especially familiar with the population they are studying—the demographics, challenges, needs, values, motivations, and goals, all of which may influence the success of recruiting and retaining participants. This manuscript reviews motivations, barriers, and strategies to recruitment and retention in clinical trials, focusing on trials in the chronic pain arena.

### Participant motivation

Many patients considering participating in clinical trials are motivated by the chance to benefit in some way. And patients want to feel reasonably confident that perceived benefits outweigh potential risks, inconveniences, and other downsides.

Surveys show that, in general, patients regard clinical trials as essential to medical progress and want to participate in them (7, 8). It also indicates that clinical trial participants are chiefly motivated by the desire to help others, personal benefit, and the perception that they are a good fit for the study (and vice-versa) (8–15). Patients are often influenced by close relationships when facing a decision to participate in a clinical trial. In a review of the medical literature on clinical trial participation among patients with chronic pain, participants ranked professional rapport with research staff among the top three motivations (along with access to treatment and altruism) for trial participation (9). Specifically, they stated that they were strongly motivated by research team members who were good listeners, empathic, trustworthy, and socio-demographically representative of the patient population (9).

A desire to help others may be directed toward those with the same condition (e.g., other patients suffering from pain), an organization or group of which they are a member (e.g., service members), the research team or institution performing the study, or medical science more broadly (14, 15). Some are motivated by “conditional altruism,” a primary desire for personal gain reinforced by an incidental benefit to others (8). For some participants, believing in the importance of the trial is a decisive element (12, 16).

Patients may see the study as an opportunity to access high-quality care, including more frequent medical attention and access to treatment and technologies that might otherwise be unavailable. Others may be motivated by curiosity or an interest in scientific research (14). Patients with chronic pain may be especially keen to participate in clinical trials investigating non-opioid complementary and alternative (CAM) treatments in the hope of reducing or avoiding exposure to addiction-forming opioid medications (14). Some patients are attracted to meeting new people, making social connections, and being involved in something inspiring and important (15). Patients also report being motivated by being a “good fit” for a study and being asked (14).

Psychological models of motivation provide a window into what moves eligible patients from “maybe” to “yes.” In a prospective study aimed at characterizing the psychological profiles of phase II and III clinical trial participants, patients who agreed to participate scored higher on metrics of self-efficacy compared to those who declined, suggesting that willingness to participate correlated with a general sense of personal empowerment or ability to effect change through one's own volition (17).

### Participant engagement

Scientists who study clinical trial engagement suggest viewing the psychology of engagement through self-determination theory (SDT). Self-determination theory posits that intrinsic motivation, i.e., the motivation that arises from one's own faculties, is a more reliable driver of behavior than extrinsic motivation (also called “controlled motivation”), i.e., motivation related to external rewards and penalties. Intrinsic motivation is characterized by three factors: autonomy (“behaving with a sense of volition, agency, and choice”), competence (“feeling able and effective”), and relatedness (“feeling connected and a sense of belonging to others”) (4).

In a recent literature review, Gamble and colleagues identified, categorized, and subcategorized successful strategies for boosting clinical trial engagement, thereby creating a kind of motivational mechanism of action blueprint for researchers (4). Strategies aimed at enhancing participant “autonomy” have included flexibility to accommodate participants' requests and scheduled reminders about study benefits and personal motivations for enrolling. An example of using flexibility to reinforce autonomy would be to modify the usual follow-up practice (e.g., leaving fewer voicemails) if requested (4).

Personal empowerment is consistent with the notion that autonomy and competence, two determinants of self-determination are facilitators of engagement (i.e., willingness to participate). Other predictors of willingness to participate in clinical trials included curiosity, social support, and lower levels of anxiety compared to trial-declining peers. These psychological attributes have been correlated with well-being (17, 18).

Patients with chronic pain may feel ambivalent about participating in clinical trials. On the one hand, many patients with chronic pain have a high disease burden and feel dissatisfied with the efficacy of available treatments, a factor associated with motivation for clinical trial participation. On the other hand, patients with chronic pain often lead complicated lives, as pain is often complicated by fatigue, insomnia, depression, anxiety, disability, and immobility, and many have comorbidities that might have the effect of lowering the desire or ability to participate (19–22).

### Is compensation a motivator?

Reimbursement to participants for costs incurred in the course of study participation, e.g., travel to and from visits, parking validation, incidental expenses, is a valuable and appropriate incentive to participation, both among economically disadvantaged populations and more broadly (23). Beyond covering costs, the practice of incentivizing recruitment and retention with financial payments is also regarded by most researchers and participants as ethical, appropriate, and acceptable. Reasonable monetary compensation, an example of an “extrinsic motivation,” is commonly viewed as an expression of gratitude for participants' time and contribution (23).

Among healthy volunteers in phase I trials, monetary compensation is a commonly cited—sometimes deciding—influence on the choice to participate (23). Although less motivating, monetary compensation may still play a role in the decision to participate in later phase trials (9, 11, 12, 23). A nested sub-study provided a window into the effect of monetary compensation on recruitment in a population dealing with pain (12). Eight months into their study, a team conducting a clinical trial on acupressure for treating dysmenorrhea added a new one-time payment to the study protocol to boost enrollment and expand the eligibility age. The changes were effective; recruitment nearly doubled.

Having created a new stratum within the study population—those incentivized by the payment and those recruited earlier who were not—researchers were able to interview participants to understand their motivations better (12). Participants in both groups reported that their decision was based mainly on their desire for pain relief, viewing the intervention as appealing and harmless, the importance of their condition to themselves and other women, and dissatisfaction with recommended treatments (typically over-the-counter analgesics) and current medical care. Two participants in the incentivized group said the stipend was a deciding factor; however, most respondents said it was not. However, for many multisession studies, participant compensation is pro-rated, not only for attending sessions but for completing research instruments e.g., daily symptom, food, and/or medication diaries. Pro-rating compensation across multiple visits reduces the possibility of inappropriately influencing someone to stay in a study to receive a lump sum payment. Escalating incentives are often used in longitudinal and/or studies with multiple follow-ups, where specific data points are essential.

Researchers concluded that while financial incentives improved recruitment, the potential for symptom relief and the desire to help others were stronger drivers in this population of reproductive-aged women (12). This comports with other research showing that, while appreciated and inspiring, monetary compensation is unlikely to be an isolated driver of later-phase clinical trial participation (10).

### Recruitment barriers

Many factors may conspire to bar patients from clinical trial participation. Patients often need to be made aware of clinical trials for which they might be eligible (7, 22, 24). Other barriers may be psychological, logistical, socio-cultural, economic, treatment-related, and/or protocol-related (8, 9, 22, 24, 25).

Researchers who study pain report the following common barriers: being understaffed and underfunded, late or limited referral from in-network and out-of-network referral partners, reluctance of pain sufferers to participate, and overestimating the local prevalence of eligible patients (22). Pain patients cite mistrust of clinical trials, fear of interventional risks, fear of inadequate treatment, too many procedures, daily stressors, and reasons related to the research team's communication of information as primary reasons for declining clinical trial participation (9).

Trust is a complex concept and warrants further discussion as it is a significant factor in participant decision-making and is commonly over-simplified. Trust (noun) is “reliance on the character, ability, strength, or truth of someone or something.” To trust (verb) is “to commit or place in one's care” or “to rely on the truthfulness” (26). Although not a guarantee, trust is a precursor to clinical trial participation (27). Trust is both a goal and an ongoing process.

Mistrust in clinical trials is commonplace, particularly among individuals with negative medical experiences and among groups (e.g., African-American, Latinx, and other racial and ethnic minorities) who have historically experienced medical discrimination and exploitation (7, 27–30). Mistrust in clinical research is often multifactorial, may be conscious or subconscious, and may be reinforced by beliefs, attitudes, and messages among peers, family members, social and mainstream media, and other community members, sometimes including, unfortunately, healthcare providers (13, 15, 24, 27). Facing a decision around trial participation, patients may host an array of concerns, including how they will be treated, what will happen to their specimens and their data, whether or not their contributions will be appreciated, and whether they are being coerced, whether information about risks of participation is being withheld (13, 27, 29). Many or all of the patients' concerns may go unexpressed.

It is critical that researchers and recruiters invite potential participants to share their questions and concerns, provide information as appropriate, and help them sort through ambivalence (13). Doubts and questions that go unaddressed allow mistrust to linger and interfere with recruitment. This is harmful, as it perpetuates disparities in the scientific knowledge base and slows the progress of science. Additionally, when patients decline participation due to unexpressed concerns, they lose access to potential benefits participants commonly report: access to high-quality care, new relationships and a sense of community, deepened understanding of their condition, and satisfaction of contributing to science (8, 10).

There is an ongoing trend among researchers and patient advocates for greater inclusivity among clinical trial participants so that the knowledge base is demographically representative of patients (16, 27–29, 31). As for many studies, recruitment goals should go beyond sufficient sample size to include sufficiently diverse representation among enrollees. Participant trust is essential to meeting these goals—trust in their referring practitioners, research teams, and the information about the study (7, 27, 28). Tools for eliciting patient concerns and helping them validate and navigate mistrust and ambivalence are shared below under *Skillful Communication*.

In under-represented populations, mistrust is often interwoven with other factors that preclude participation—lack of awareness of clinical trials, study design, logistical challenges, challenges related to social determinants of health, family issues, and communication barriers. These recruitment challenges underscore the importance of using multi-pronged, culturally sensitive strategies to meet recruitment goals (7, 31).

### Mitigating barriers and improving recruitment

Studies comparing the efficacy of specific recruitment strategies are few. In a Cochrane meta-analysis of comparative recruitment studies, only two approaches could be said with high-level evidence, even modestly, to improve recruitment: open-label study structure and following-up mailed invitations with phone calls (32). In the absence of consensus around best practices, and as significant variability exists in study goals, types, and target populations, we are left to mine published reviews and individual trial post-mortems to identify themes and methods that might offer some direction for future efforts. Doing so uncovered five overarching themes or strategies for reducing barriers and increasing recruitment in clinical trials: patient-centric design, making trials more convenient and accessible, clear communication, sociocultural representation, and a multi-pronged strategy rooted in strong relationships with community healthcare providers.

## Patient-centric design

There is a steady trend toward patients becoming increasingly empowered, organized, and involved in their care, including playing more significant and diverse roles in clinical research. Perhaps the best way to ensure adequate recruitment and retention is to design clinical trials around patient preferences. Patient-centricity can be described as reframing the patient's role from the passive receiver of prescriptive, top-down care to an active collaborator whose experiences, perspectives, and inputs inform all aspects of clinical care and research related to their condition (33). The patient-centricity movement emerged at the confluence of several phenomena—increasingly well-educated and well-connected patient groups, technological advances that allow for remote data gathering and care delivery, and a growing appreciation among both patients and clinicians for the limits of the conventional biomedical model toward understanding and addressing patients' persistent problems (34).

One aspect of patient-centricity is using qualitative data from patient interviews to inform the design and implementation of clinical trials. During the pre-planning stages of the trial, surveying patients about their goals, values, struggles, and lifestyles can inform decision-making related to research priorities, outcomes, recruitment and retention strategies, protocol details, and plans for disseminating findings and implementing results (35). Questions about values and struggles might include, “What bothers you the most about your condition? How does your condition affect your daily life? What would help you better manage your condition? What do you find most objectionable about your current treatments? What is one thing you would change if you could?”

Questions about logistics and lifestyle might include, “What would make you want to participate in a study? What would make it workable for you? Would you prefer on-site visits, telemedicine, or a combination of both? How many visits would you be willing to attend over what period of time? What might interfere with your ability to participate? Do you like or dislike the idea of being paid to participate? How would you like to be informed about study results?”

One might also inquire about media consumption (e.g., radio, TV, magazines, podcasts) and community memberships/activity (e.g., religious, medical, civic) to inform study advertising strategy with questions such as, “Where do you get your news? What websites do you visit regularly? Have you ever participated in a clinical trial before? If so, how did you hear about it?” Patient surveys may be conducted as focus groups or via questionnaires distributed in person, online, or via email (31).

The value of patient-centric study design to recruitment and retention is multifold. Designing trials around patients’ insights demonstrates respect for their contributions and prerogatives, acknowledges their role as principal stakeholders, assuages fears around exploitation, and helps to engender trust in the process. Patient-centered trials have been shown to improve patients' satisfaction, recruitment speed and success in meeting targets, enrollment diversity, participant retention, and cost-efficiency (8). Patient-centric design encourages partnership with participants as members of the research team and can enhance engagement (36).

Patient-centric also means designing trials that are realistic and easy to implement. Complex protocols, even basic RCT requirements such as randomization and blinding, may be disincentivizing (13, 24). A Cochrane meta-analysis of research into methods and factors in clinical trial recruitment found high evidence that recruitment efforts were 10% more successful for open trials compared with blinded, placebo-controlled (32).

The chance to be randomized to a placebo group or less active comparator may be particularly discouraging to patients with significant or longstanding disease burdens, such as patients with chronic pain and those whose rationale for participating centers on access to investigation drugs or procedures. Recruitment staff should be prepared to explain the scientific method in plain language and offer workarounds for objections. For example, a reasonable solution is to provide active treatment to those randomized to placebo and wait-listed arms after the study ends, thus ensuring universal investigational treatment access to participants (37). Alternatively, incorporating open-label or crossover phases into the trial would serve a similar purpose and generate additional data (38). Another alternative approach to randomization is optional blinding based on patient preferences (25). Investigators must weigh the costs of additional visits (staffing, space, and financial) with potential benefits on participant and staff morale, recruitment, and retention.

## Making trials more convenient and accessible

In surveys, patients report a preference for clinical trial structures that are convenient and flexible in design so that the burden of participation is low (24, 25). Features such as proximity to the study site, fewer in-person visits, and reimbursed costs are likely attractive to potential participants (39). However, different patient populations want different things; therefore, gathering insights from the population being studied is essential. For example, most preferred a shorter study duration in a survey sent to patients with two or more chronic diseases. However, the subgroup of patients with chronic pain significantly preferred a longer study duration (25). The distinction was not explained; however, it might reflect a high priority among patients experiencing pain for symptom relief that investigational treatments might afford.

Several studies have asked patients what they would prefer in hypothetical clinical trials. In one, patients with chronic pain stated that minimizing invasive lab tests and the ability to continue current pain medications during the trial were the two most important aspects of a desirable clinical trial (39). These findings suggest that patients living with pain may be particularly motivated to avoid additional pain incurred during study participation and to protect pain control gains already achieved. In the same survey, monetary compensation and fewer in-person visits were also ranked as essential incentives, contradicting the above study findings (39).

In a third study, patients with chronic diseases were presented with hypothetical clinical trial scenarios. They were asked to share their preferences for in-person, at-home via video, or a mixed in-person and at-home approach (40). The mixed approach was the most popular. Patients also reported a desire for greater choice (in study organization and appointment times), flexibility (appointment times), convenience (combining trial follow-up with routine care visits), and respect (designated waiting areas for clinical trial participants, not being made to wait, reimbursement for parking and transportation) (40).

Investigators seeking to optimize recruitment and retention should design trials that align with participant preferences. This may include allowing for some flexibility in requirements when it may improve engagement without compromising the study's integrity. Depending on the nature of the study, solutions for balancing participant and research needs might include (4, 35, 38, 41).

• expanded or flexible appointment times, such as evening or weekend hours for participants who work or go to school during regular business hours
• offering home follow-up visits to accommodate participants with mobility or transportation barriers
• scheduling phone calls at convenient times
• flexibility in scheduling tests
• on-site childcare
• reimbursement for travel, parking, and childcare
• allowing participants to integrate certain study requirements (e.g., follow-up visits, assessments) into their routine clinical care
• use of digital technology for gathering patient data

Surveys also point to the advantage of integrating clinical trial visits with routine clinical care when possible (35).

## Skillful communication

One of the main ways researchers can improve study engagement is enhancing professional skills in clear communication (42). Patients report that communication factors into the decision to participate in clinical trials (7, 43). It is useful to examine the intersection of communication and decision-making and consider methods most appropriate for communicating with potential participants.

It is reasonable to think that, among eligible patients who are otherwise inclined to participate, those who are trusting vs. mistrusting self-sort into participant and nonparticipant groups, respectively. But it is arguably not that straight-forward. One theory posits that trust and mistrust are not static but dynamic states that can coexist relative to a single topic in a single individual. Ambivalence—an uncomfortable internal state that arises from holding contradictory opinions, plays an important role in the complex fluctuations of trust and mistrust (43). Individuals are faced with a decision using various strategies in an attempt to reduce ambivalence, such as defaulting to the stronger or more long-standing position with little thought (dominance) or avoiding the decision altogether (avoidance). Alternatively, one might manage ambivalence using “holism,” a slower process that involves remaining open to both positions' positive and negative attributes, placing pros and cons on one side or the other until the scale in their mind tips definitively in one direction (43).

When approached with the opportunity to enroll in a study, patients who are not ambivalent (i.e., clear-minded) around the choice and those managing ambivalence using dominance or avoidance can be expected to decide quickly, either for or against participating. On the other hand, patients who hesitate are likely processing internal conflict or competing values (i.e., strong interest both for and against participating) are managing using holism.

What's important about this in terms of clinical trial recruitment is that if recruitment teams recognize hesitation as a normal response to strong ambivalence and recognize ambivalence as containing strong interest (that is, being actively opposed by valid concerns), they can communicate in ways that reduce cognitive barriers and help patients manage their ambivalence and arrive at an informed choice (42, 43). Under these circumstances, what helps are free choice, information, safe relationships, and a lack of ambivalence in people of influence (43).

Based on this understanding, a conceptual framework for effective recruitment conversations includes (30, 42, 43):

• seeking to build trust and a sense of legitimacy
• creating an unpressured, nonjudging space where patients feel free to choose
• being genuine, honest, and transparent while holding an unequivocally positive orientation about participation
• providing information about the trial purpose, objectives, procedures, benefits, and risks in clear, easy-to-understand language
• being prepared to answer questions and address objections

Investigators might find it valuable to employ a method of conversation called “motivational interviewing” (MI) used in health and wellness coaching and other fields to help individuals increase intrinsic motivation and resolve ambivalence (44). Motivational interviewing, the basics of which may be learned in a brief training, is used in medicine for many purposes, e.g., to improve medication adherence and medical outcomes (45, 46). Motivational interviewing uses open-ended questions, active listening, and other dialogue skills to help patients express, validate, and explore their views and feelings to effectively process ambivalence and make decisions in their best interest (43). An open-ended question cannot be answered by a simple “yes” or “no.” Open-ended questions typically start with “how” or “what.” The simplest example of MI is to ask, “What information would help you decide?” “What else is on your mind about this?”, or “How can I best support you in this decision?” Notice that these questions invite the interviewee to share their perspective and have the effect of forwarding rather than terminating the conversation.

Further, many participants enter clinical trials interested in seeing their contributions lead to real-world impact (11, 35, 47). A plan for following up with participants and sharing results shows respect for their contributions and represents a meaningful close to the contract.

Asking, listening, and providing information enable patients to assess the merits of participation for themselves and come to their own conclusion. Other trust-engendering communication strategies sharing testimonials of past participants of similar or preceding trials, having senior investigators reach out to participants, disclosing study funding, and sharing information via lay community members, which is discussed below (42).

## Expand sociocultural representation and reach

Black, Hispanic/Latinx and other minority groups are underrepresented in clinical research (28–30). Strategies for improving clinical trial participation among under-represented groups discussed above include efforts to engender trust, improve communication, assist decision-making, and design trials better suited to patients' needs. Other pivotal strategies include bridging language barriers (e.g., employing bilingual staff, using bilingual forms and materials, hiring independent interpreters) and using socioculturally respectful approaches to recruitment. However, the former may be impractical or cost-prohibitive (30, 31, 38).

Surveys show that many Hispanic/Latinx and African American-identifying patients feel more comfortable with individuals sensitive to their culturally specific needs. This may mean employing members of their own communities to play central roles in introducing studies (7, 28, 30, 48). In a stroke-prevention clinical trial, researchers conducted interviews with potential candidates in an African-American population to understand better hesitations around participating. They learned that patients were largely dissatisfied with the way they were approached (7). As a result of their study, researchers proposed that information about clinical trials might be better received in African-American communities if disseminated through community-based resources, e.g., speaker's bureaus, church groups, and health fairs. Other recommended strategies to improve trust and uptake included creating a community-based advisory panel to facilitate recruitment, having more culturally sensitive and representative research personnel, having more dedicated African-American personnel, and alleviating economic and logistical barriers with measures such as travel reimbursement and flexible hours for working individuals (7).

The value of harnessing community ties to boost recruitment was shown in a separate study, which aimed to invigorate recruitment of African American and rural-dwelling palliative care patients. Potential participants were introduced to the study by a community member of the same race in partnership with a research team member. Fifty-nine percent of those approached by the team included a community member, compared to 13% of those approached by the research coordinator alone (48).

Social distancing requirements of the COVID-19 pandemic accelerated a growing interest among researchers in the decentralization of clinical trials and the use of digital data-gathering tools to reduce costs, extend reach, and reduce participation inequities (29, 49). It has been postulated that reduced geographic constraints and greater overall convenience would be a boon for clinical trial participation among diverse populations. Decentralization represents a radical departure from traditional institution-based clinical trial methodology, rife with its challenges, including the digital divide, the need for new infrastructure, and significant limits to the ability to perform procedures, deliver interventions, and monitor patients (50).

Still, the momentum toward using digital technologies to improve clinical trial reach and efficiency is undeniable. Innovative strategies with digital or automated components are increasingly heralded as central to efforts to improve access to clinical trials across genders and races (51). A review of digital tools used in clinical study recruitment showed that social media, internet sites, email, and tv/radio were the most popular platforms. Online advertising methods included banners, search engine optimization/Google AdWords, online press releases, electronic newsletters, podcasts and webinars, and online community notice boards. Blended approaches included printed flyers and mail-outs that directed recipients to a website for more information and screening questionnaires (41).

## Multi-pronged recruitment approach

Research points to the value of employing a multi-pronged approach to meet recruitment goals, starting with relationships with local medical colleagues (52, 53). Patients prefer to be recruited by their primary care physicians or community members with whom they have trusted relationships (8). Primary care and community-based providers are often rich sources of referrals for clinical trials, especially those with high levels of trust in medical research and who have previously referred patients for clinical trial participation (38, 54).

Patients commonly report dissatisfaction about the disconnect between the delivery of clinical care in the community and research and want more integration of the two (4, 10). Mechanisms for linking the administration of clinical trials with the delivery of routine or specialty healthcare should be considered, such as educating patients about clinical trials during routine outpatient visits (while being mindful of optimal timing to not overwhelm sick patients) (24, 35). Further, although additional coordination and possibly incentivization might be necessary, efforts to weave elements of protocol delivery, e.g., trial follow-up examinations and assessments, into patients’ pre-existing healthcare engagement patterns would reduce the burden and likely motivate participation (35, 55).

Researchers should seek to maintain high-caliber professional relationships with health care providers and community programs near the study site, keep them apprised of planned trials, and support recruitment via in-person education, informational sessions, printed flyers, waiting room posters, and other tools (24). Other potential referral sources include databases from clinical laboratories, blood banks, government employees, and related clinical research, and outreach mechanisms include the press, media, postal mail (with follow-up phone calls to nonresponders), email, and online sites (24, 32, 52, 56).

### Participant retention

If recruitment depends on creating a mutually agreeable contract between research teams and participants, then retention depends on fulfilling that contract throughout the trial and working to build upon participants' initial motivation. Factors that have been reported to interfere with participant retention in clinical trials include: experiencing adverse events, lack of improvement, being assigned to a placebo arm, fear of study procedures, uncomfortable or extra procedures, trial conduct-related issues, waning motivation, lack of social support, lack of support from family physician, high study demands or inconveniences, negative media publicity, life change (e.g., change of residence or need to care for a family member, changed health status), and poverty-related issues (4, 24, 57). Research participants are real people with real struggles, some of which may preclude ongoing participation at some point in the trial. Some degree of attrition may be inescapable. In other instances, however, study withdrawal may be preempted or mitigated by incorporating engagement-boosting strategies into study procedures and staying vigilant for dropout risk-signaling behaviors.

### Strategies supporting engagement and retention

Strategies that enhance “competence” include any action that helps participants gain knowledge related to their participation and combats potential feelings of being neglected or uninformed. Initiatives have included informational and educational events about their disease or their role in the scientific process. A simple strategy that supports participant autonomy and competence is sharing study results with participants when available. Many participants enter into clinical trials interested in seeing their contributions lead to real-world impact (10, 11, 35, 47). Following up with participants and sharing study results and publications shows respect for participants' contributions.

Research teams report using various measures to enhance “relatedness” between participants and research staff (1, 4). Interpersonal interactions and attitudes that allow participants to “feel seen” and appreciated improve relatedness and, hence, engagement. Examples include language concordance, sociocultural sensitivity, fostering rapport, and establishing a safe, supportive, approachable, non-patronizing atmosphere (4, 8, 28). Initiatives aimed at “relatedness” have included newsletters sharing testimonials of former participants or study updates, social media engagement, engagement of participants' family and friends, and maintaining consistent personnel-participant pairings so that a relationship can develop over time (4). Some have found that “personal touches”—such as personalized letters, greeting cards (e.g., holidays, birthdays, thank you, condolences, or get well for family members), or gifts (e.g., framed pictures of participants with staff help)—support engagement (4, 58).

Staff training in protocol delivery, professional conduct, skillful communication, and empathy facilitates professional behavior and “relatedness” and is valuable for participant engagement (4).

### Organizational competence

Extrinsic motivators for engagement include basic measures demonstrating an interest in participants' experience and organizational competence (4). Physical spaces for meeting with and delivering trial interventions to participants should be clean, tidy, aesthetically pleasing, well-maintained, and functional for all individuals, including patients with impaired mobility or disabilities (57). Corridors should be sufficiently wide and kept clear of obstructions; wheelchair access and physical supports (e.g., for climbing on and off treatment tables) should be in place for individuals who need them. Proper lighting and way-finding signage support the ease and safety of navigating clinical spaces. These and other basic considerations, such as a separate designated waiting space for trial participants keeping appointments and starting them on time, demonstrate respect for participants' contributions and support ongoing engagement (40).

Various methods for reminding participants of appointments may improve study efficiency and reduce attrition (4, 32, 57). These might include appointment cards or calendars, mailed postcards or letters, phone calls, text messages, and emails with follow-up mechanisms triggered by failure to confirm.

### Mitigating drop out

Team members should stay vigilant for signs that a participant might be struggling with engagement and seek to understand and assist them in finding a resolution within appropriate limits (4). Signs of medium to high risk for study withdrawal include frequent cancelations without rebooking, failing to attend appointments (“no-shows”), being unresponsive to efforts to contact them, reluctance to schedule an upcoming session, or voicing dissatisfaction, waning interest, new barriers to attendance or a desire to drop out (57).

Working with human subjects is inherently variable and somewhat unpredictable. To address suspected or imminent drop-out, researchers must attempt to accommodate participant needs (e.g., through supplemental attention, information, flexibility) while avoiding introducing bias into the system (59). Targeted retention strategies, as described above, are sometimes necessary (59, 60).

## Conclusion

Recruitment and retention are established challenges that can make or break clinical trials. Success lies in seeking to understand and optimize the experience of their study population, employing targeted outreach and engagement strategies, and adapting as necessary to accommodate participants' needs, when necessary and possible. Researchers optimize engagement by considering recruitment and retention strategies before study design, considering participant perspectives, and embracing patient-centricity. Helpful strategies include using multi-pronged recruitment avenues and strategy, harnessing established trusted relationships between community providers and potential participants, employing staff or lay community members representative of patient populations, helping patients process and overcome ambivalence through skillful communication. Steps to create a professional environment, reduce barriers related to inconvenience and cost, and create an overall positive patient experience can be expected to optimize engagement and support all aspects of clinical trial execution see Table 1.

**Table 1:** Principles and strategies for Recruitment in clinical trials.

I. Embrace patient-centricity
• Interview representative participants during pre-design/design phases
• Select research topics and objectives based on what matters to patients
• Design and implement participant acceptable trials
II. Design trials more convenient and accessible
• Cover participation costs (transportation, parking, childcare)
• Extended or flexible office hours/visit times
• Accessible study site; home visits for disabled
• Limit visits/tests to those that are essential
III. Skillful communication
• Professional rapport and trust is central to collaboration and engagement
• Trust and distrust are moderated by ambivalence
• Help participants manage ambivalence
• Ask open-ended questions, listen and inform (rather than persuade)
• Be genuine
VI. Expand socioeconomic reach
• Set both sample size and diversity enrollment goals
• Inclusive eligibility criteria
• Hire multicultural, multi-lingual research staff
• Bridge language barriers
• Employ demographically matched, community-based assistance
• Anticipate need for additional time and resources to support involvement of diverse populations
V. Use multi-pronged approach
• Strong relationships with community providers
• In-person education
• Combine printed and digital advertisements

Investigators should be especially sensitive to the needs of patients experiencing debilitating symptoms, including chronic pain, be amenable to their inputs, and be prepared to support engagement with behavioral and pragmatic strategies to boost relatedness, autonomy, and competence.
